# Young woman with Guillain‐Barré syndrome and cervical transverse myelitis—A new GBS variant, not coincidence

**DOI:** 10.1002/ccr3.2818

**Published:** 2020-04-21

**Authors:** Jenna Gharzeddine, Brian Renner, Natalie Wassall, Kristen Tran, Antonio Liu

**Affiliations:** ^1^ Department of Neurology White Memorial Medical Center Los Angeles CA USA; ^2^ Department of Neurology California Hospital Medical Center Los Angeles CA USA; ^3^ Department of Neurology Cedars‐Sinai Health System Los Angeles CA USA

**Keywords:** Guillain‐Barré Syndrome, MRI, neurodegeneration, neuroimmunology, neurology, transverse myelitis

## Abstract

A case of antibody proven Guillain Barré Syndrome in a previously healthy young female with extra clinical features, scans, and physical exam findings consistent with cervical spine and cervical medullary junction myelitis, together a new variant to consider.

## Graphical abstract

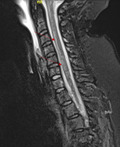

## 1 INTRODUCTION

When multiple similar comorbidities overlap from seemingly different etiologies, it raises the question whether there is a connection or even a common etiology. We present a case of antibody‐proven Guillain Barré Syndrome (GBS) in a previously healthy young female with extra features, scans, and physical exam findings consistent with cervical spine and cervical medullary junction myelitis. The case presented here exhibits a case of new variation in GBS indicating further that it is a spectrum of autoimmune complications and that other neurological degenerative disorders, transient or permanent, could have a common pathogenesis.

Guillain‐Barré syndrome is a rare disease primarily affecting the peripheral nervous system (PNS), often sparing the central nervous system (CNS).^1^ It generally presents as an ascending areflexic motor weakness without incontinence while sparing sensation,^2^ though there have been many variants described in literature which have varied presentations.^3^ The most common form of GBS (also known as acute inflammatory demyelinating polyneuropathy—AIDP) is rapid ascending paralysis.^4^ Variants of GBS include, but are not limited to, acute motor axonal neuropathy, Miller Fisher variant (MFv), and pharyngeal‐cervical‐brachial variant. Other variants combine features of GBS and central nervous system deficit,^5^ like Bickerstaff's brainstem encephalitis (BBE), cross over MFv‐BBE and a few cases of concomitant GBS and transverse myelitis. Another neurological inflammatory disorder is transverse myelitis, which most commonly presents as inflammation in the spinal cord leading to marked CNS deficit.^6^ This deficit can vary in presentation though is frequently seen as a rapid onset of motor weakness, bowel/bladder dysfunction, clearly defined level of spinal involvement and the potential for alterations in sensorium. It is diagnosed using specific criteria of clinical presentation, bilaterality, and MRI T2‐weighted gadolinium‐contrast imaging or elevated IgG in CSF studies.^7^, ^8^

**Figure 1:**
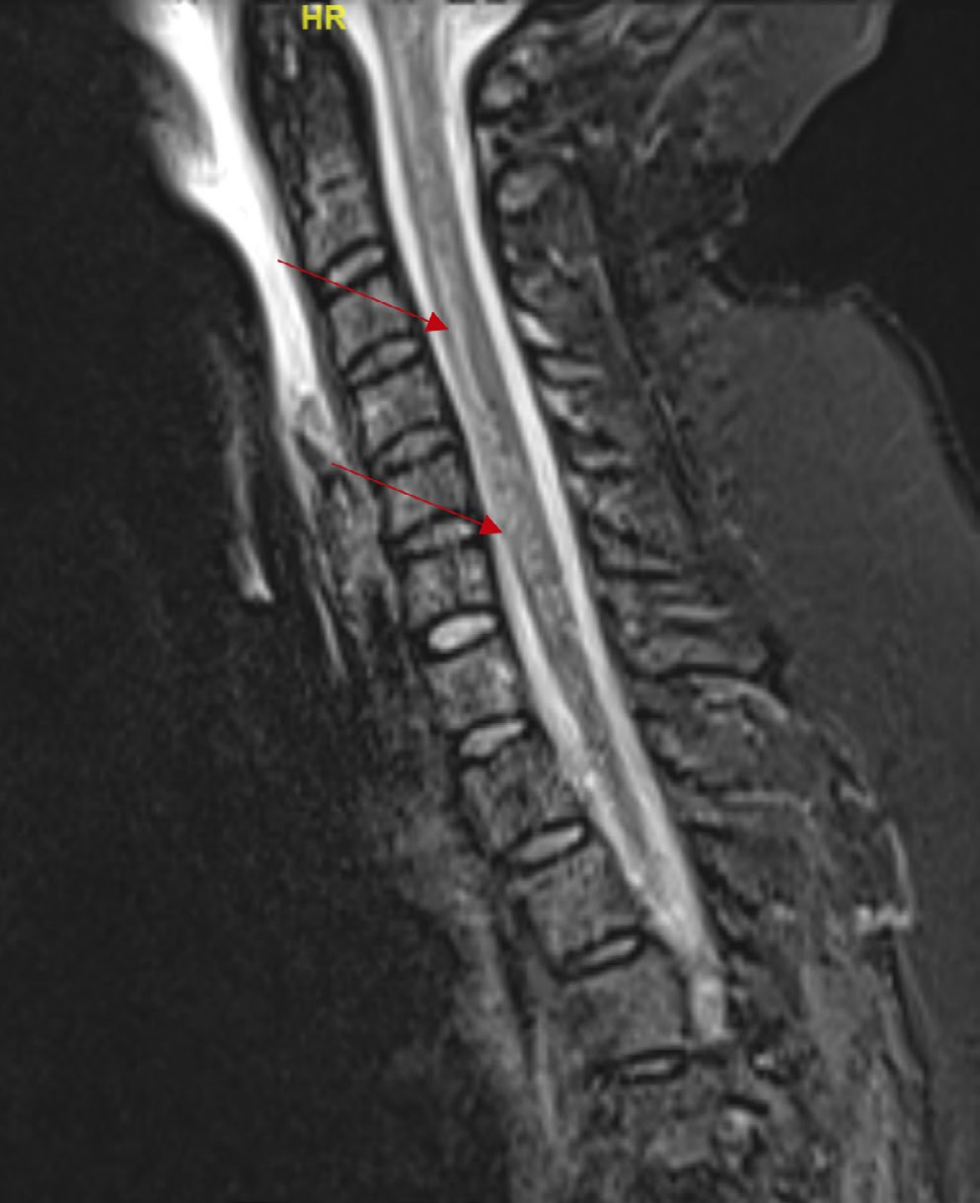
Sagittal T2 MRI abnormality seen within the spinal cord in high cervical area. Red arrows indicate hyperintensity shown on imaging

**Figure 2:**
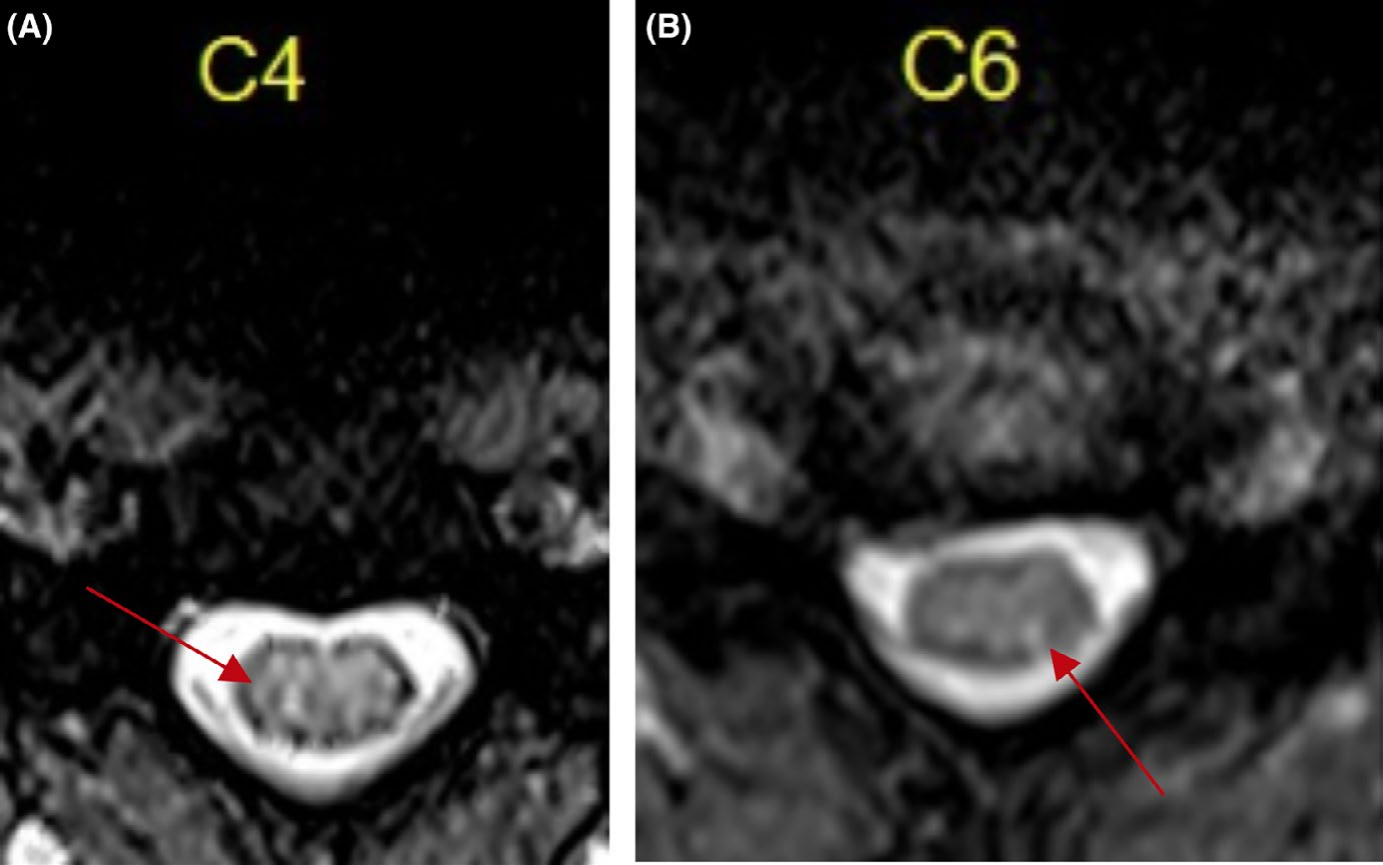
Axial T2 MRI gradient axial shows diffuse abnormality at (A) C4 and (B) C6 levels

We present a case of a young female patient with typical ascending areflexic weakness consistent with GBS who also has high cervical and cervicomedullary junction findings on examination with the patient endorsing urinary incontinence, sensory loss, and vertical nystagmus. GBS was confirmed by the presence of ganglioside antibodies, and TM was confirmed by the presence of high spinal cord lesions on her MRI scan of cervical spine seen in multiple planes (Figures 1 & 2) while MRI brain images were found to be negative for pathology. The patient received plasma exchange to treat the GBS and high dose corticosteroids to treat the TM. She subsequently experienced remarkable improvement prior to being transferred to long‐term inpatient rehabilitation.

We propose that the observed simultaneous findings of GBS and TM are not coincidental; the cases of GBS which have an associated cord signal on MRI can be viewed as a new variant of the disease. On literature review, we found fewer than 30 reports documenting the confirmed coexistence of TM with GBS^9^; we submit this case as an addition to the collection of case reports that supports this new variant of GBS.

The extremely rare pairing of GBS alongside cervical TM has recurred in cases worldwide,^5^, ^10^ considered a coincidental finding.^11^, ^12^, ^13^ To date, these two illnesses are considered two separate entities, despite similarities in disease pathophysiology.^14^ The continued reappearance of the rare GBS and TM duo points to a new variant of Guillain‐Barré syndrome as well as raised clinical suspicion when overlapping symptoms are present.^11^ The case presented here exhibits a case of new variation in GBS indicating further that it is a spectrum of autoimmune complications and that other neurological degenerative disorders, transient or permanent, could have a common pathogenesis.

## 2 CASE PRESENTATION

A 40‐year‐old, previously healthy, obese female patient developed lower extremity numbness and tingling four days prior to admission. There was no associated weakness or progression of her numbness until the day prior to admission. Preceding these symptoms, she had developed left facial weakness one week prior and was previously diagnosed with Bell's Palsy; she had no recent illnesses nor recent vaccines. On examination, she had left lower facial weakness with minor forehead involvement. Strength in her upper and lower extremities were 4/5 and 3/5, respectively. No deep tendon reflexes were elicited, and Hoffman's sign was negative. Sensory examination showed bilateral decreased sensation to light touch and ice below her neck, about C5 and below; finger and toe sensations were equivocal. Extraocular movements were intact, and nystagmus was absent on presentation. There was no bowel and bladder involvement. She denied headache and had normal mentation.

On day two of admission, her weakness and numbness progressed. Her motor examination was 2/5 in all extremities without elicited reflex at any location. She developed bilateral ptosis while extraocular movements remained intact without nystagmus. Urinary incontinence developed, and she showed signs of early respiratory failure.

On day three, her ptosis continued to worsen, and she began to develop ophthalmoplegia. The patient also developed prominent nystagmus in all directions. She had upbeat nystagmus on up‐gaze and downbeat nystagmus on down‐gaze. She developed respiratory failure requiring intubation and mechanical ventilation.

Prior to day three, the patient's vital signs had always been within or near normal limits. Maximum temperature throughout the hospitalization was 99.2F. Her BP was at its maximum on admission at 167/78 and subsequently dropped to 130s/60s for the duration of her admission. Serum white blood cell count was 15.6 on admission and dropped down to 9.0 at lowest count. No other chemistry abnormality was seen on laboratories. Vitamin B12 was 1324, TSH 2.6, and HgbA1c 5.7. Aquaporin‐4 antibody was negative. Her CSF had 0 white blood cells, 1 red blood cell, elevated protein to 114, and glucose of 72. West Nile titer, VDRL, cryptococcus, and coccidioidomycosis titers were negative. HIV was also negative. MRI scan of head was negative while MRI scan of C‐spine showed ill‐defined T2 signal abnormality within the cord, extending all the way to, and likely above, the cervicomedullary junction. MRI scan of C‐spine was repeated twice more over the following three weeks and showed minimal to no change from baseline abnormality (Figures 1 & 2).

Her symptoms continued to progress during the first five days of admission. At most severe presentation, she was completely quadriplegic with motor strength at 1/5 in all extremities with completely absent reflex. Her sensation loss was severe in all modalities but was never absent. The patient's ocular symptoms progressed to complete ptosis with severe ophthalmoplegia.

On the Guillain‐Barré serum panel, the antibody to ganglioside N‐acetylgalactosaminyl GD1a (anti‐GD1a) was positive at 175. The antibodies to GM1, GQ1b, and other entities on the panel were otherwise negative. Following symptom progression and positive blood titer to anti‐GD1a, the patient received five days of plasma exchange. A course of IVIG was also given after plasma exchange. The patient also received 5 days of high dose solumedrol. She began to improve approximately at day seven of admission and was extubated on hospital at day eight.

The first deficit to resolve was the vertical nystagmus. By the time she was transferred out of ICU, the strength of her extremities recovered to 4+/5 in the upper extremities and 4‐/5 in the lower extremities. Deep tendon reflex was never present during hospital course nor during recovery. She was transferred to an acute rehab unit after 20 days of hospitalization for continued management and rehabilitation.

A phone follow‐up interview exactly one year after discharge revealed that the patient was able to walk normally without an assistive device. She has no incontinence and can walk up to several blocks, though she fatigues easily. She has no double vision but occasionally slurs her speech. Additionally, she has returned to work part‐time.

## 3 DISCUSSION

Guillain‐Barré syndrome is a collection of mostly peripheral acute demyelination syndromes; its incidence is quite rare—generally stated to be an incidence of 1‐2 in 100 000 worldwide^15^—and has been associated with several specific HLA immunotypes in recent studies.^16^, ^17^, ^18^, ^19^, ^20^ Beyond the most common presentation of AIDP are the infrequently seen variants of GBS.^5^ The second most common GBS variant is acute motor axonal neuropathy which is associated with poor outcomes.^20^ Other variants include Miller Fisher variant (MFv), pharyngeal‐cervical‐brachial variant, and Bickerstaff's brainstem encephalitis variant (BBE). BBE has brainstem involvement clinically and radiologically on MRI. Furthermore, there are reports of crossover where the MFv‐BBE variants have been found to overlap.^21^ Although rare, AIDP with concomitant myelitis has been described.^11^, ^12^, ^13^ A case report from Brazil details an elderly patient with GBS and a below‐thoracic TM, which the author suggests is “concomitant.” Treatments aimed at reducing immunological response are ideal in both scenarios individually. These and other previous authors believe these were coincidental findings; we believe the coexistence should be viewed as a new variant of disease.

This patient presented with progressive signs and symptoms of GBS during the first day and second day of admission. The patient's facial droop on presentation was initially mistaken for Bell's palsy and likely was the first sign of disease. However, by day three of admission the differential diagnosis evolved as signs and symptoms of a cervicomedullary junction lesion surfaced—including sensory level, urine incontinence, and vertical nystagmus. Using Brighton's Criteria, the patient is classified as category II with positive presenting symptoms, time of onset, CSF cytology, and absence of alternative diagnosis, though lacking EMG Nerve Conduction Test data precludes meeting category I of the criteria.^23^ The primary disease process of the patient's presentation is likely GBS; a continuous lack of elicited deep tendon reflex and elevated serum antibody to GD1a strongly supports the diagnosis. A cervicomedullary junction lesion would not present with ptosis and ophthalmoplegia as did this patient. Electrophysiological findings can be normal, especially in GBS variants.^24^ The pathologic development of cervicomedullary junction abnormality was apparent from both clinical symptoms and supporting MRI findings.

We suggest that cases of GBS which have an associated cord signal on MRI can be viewed as a new variant. The case presented above is not consistent with the Miller Fisher variant due to minimal extraocular movement issues aside from prominent vertical nystagmus, and maintenance of full sensory response.^25^ The case was also not consistent with Bickerstaff encephalitis as the patient had no encephalopathy. In addition, further imaging findings on MRI were not consistent with significant brain stem involvement and the patient maintained full preservation of mental status.^26^ The patient did not fit the clinical picture for the pharyngeal‐cervical‐brachial variant because the cranial nerves were involved and she was quadriplegic as well as had findings of myelitis on MRI.

Due to the patient's presentation of classic ascending GBS and cervical spine myelitis on MRI, a crossover of Miller Fisher and BBE was excluded as a diagnosis. Although vertical nystagmus has been described in the Miller Fisher variant of GBS in some cases, the authors believe the vertical nystagmus is due to cervicomedullary junction involvement. We propose that our patient with GBS and high cervical myelitis involving the cervicomedullary junction is not a coincidence but a new variant of GBS which has the classic features of AIDP and concomitant TM features of autonomic and bilateral motor deficit without cord compression with an elevation in CSF pleocytosis, lesion findings on MRI, and a progression of disease in less than 21 days.

The immunological role in polyneuropathies is a topic of continued research and elucidation that requires a mixture of clinical acumen, patient history, imaging modalities, electrophysiology, serum studies, and genome analysis for definitive diagnosis. It has been shown that there are subtypes of HLA alleles which are more predisposed to these immune‐mediated disorders^27^ and that the role of early diagnosis must be emphasized to reduce both short‐ and long‐term damage to the patient. Additionally, recognizing physical examination findings and correlating them with their associated neurological area of potential deficit can mean more quickly honing diagnostic specificity and treatment before more costly or time‐consuming testing and analysis is completed to confirm the disease. In specifying a disorder as a mixture of symptoms from a single etiology, patient care can be improved, and hospital stay, and medical costs can be reduced all the while improving patient prognosis of return to full function.

## CONFLICT OF INTEREST

None declared.

## AUTHOR CONTRIBUTIONS

JG, first author, patient seen with AL; literature review, manuscript preparation. BR, second and corresponding author, literature review, manuscript preparation and editing, significance next to JG in submission. NW, research assistant under JG contributed to research review. KT, research assistant under JG contributed to research review. AL, PI, attending physician, recognition of patient finding and proposition of new variant illness.
